# Divergent risk of SARS-CoV-2 infection, severe COVID-19 and mortality across psychiatric disorders: analysis from electronic health records in Catalonia

**DOI:** 10.1192/j.eurpsy.2024.557

**Published:** 2024-08-27

**Authors:** A. Monistrol-Mula, M. Félez-Nobrega, I. Giné-Vázquez, J. M. Haro

**Affiliations:** ^1^Epidemiology of mental disorders and ageing, Institut de Recerca Sant Joan de Deu, Sant Boi de Llobregat, Spain

## Abstract

**Introduction:**

People with psychiatric disorders are particularly vulnerable to SARS-CoV-2 infection and its associated complications. However, current literature show that not all psychiatric disorders are equally vulnerable to COVID-19.

**Objectives:**

This study aimed to assess whether individuals with distinct psychiatric disorders exhibit different risk of SARS-CoV-2 infection, COVID-19 hospitalization, and mortality.

**Methods:**

We conducted a case-control study using data of electronic health records from Catalonia. Cases included adults with a hospital admission between 2017 and 2019 for non-affective psychosis, bipolar disorder, depressive disorder, stress-related disorders, neurotic/somatoform disorders, and substance misuse. These were matched to patients without a diagnosis by sex, 5-year age band, and living area. Outcomes included SARS-CoV-2 infection, hospitalization, and COVID-19-related death up to December 2021. Logistic regression analysis were employed to test the association between the six groups of psychiatric disorders and COVID-19 outcomes, controlling for age, sex, smoking, being in a nursing home, and physical comorbidities.

**Results:**

785,378 subjects were included. Preliminary findings showed that patients diagnosed with psychosis and bipolar disorder had lower risk of infection [OR: 0.85 (95% CI: 0.79-0.92), *p*<0.001; OR: 0.84 (95% CI: 0.76-0.92), *p*<0.001], whereas individuals with stress-related and neurotic/somatoform disorders had higher risk of infection [OR: 1.08 (95% CI: 1.04-1.14), *p*<0.001; OR: 1.06 (95% CI: 1.03-1.10), *p*<0.001]. People with depressive, stress-related, and neurotic/somatoform disorders had lower risk of COVID-19 hospitalization [OR: 0.87 (95% CI: 0.78-0.97), *p* = 0.01; OR: 0.71 (95% CI: 0.61-0.84), *p*<0.001; OR: 0.67 (95% CI: 0.60-0.76), *p*<0.001]. In line with these results, individuals with stress-related disorders also experienced lower mortality [0.49 (95% CI: 0.33-0.70), *p*<0.001]. Conversely, people with psychosis, bipolar disorder, and substance misuse exhibited higher risk of COVID-19-related death [OR: 2.9 (95% CI: 1.68-3.1), *p*<0.001; OR: 1.95 (95% CI: 1.30-2.81), *p*<0.001; OR: 1.82 (95% CI: 1.49-2.20), *p*<0.001].

**Conclusions:**

We found different risks of SARS-CoV-2 infection, COVID-19 hospitalization, and COVID-19 mortality for psychiatric disorder groups. Differences in vulnerability to COVID-19 among people with psychiatric disorders might be explained by factors such as shared living facilities, physical comorbidities, psychotropic medications, and difficulties in accessing high-intensity medical care. Special attention should be directed towards individuals with psychosis, bipolar disorder, and substance misuse.

**Disclosure of Interest:**

None Declared

